# Activity of HOCl-generating e-bandage with clinically available hydrogels against *Staphylococcus aureus* and *Acinetobacter baumannii* biofilms

**DOI:** 10.1128/spectrum.01762-25

**Published:** 2025-09-18

**Authors:** Eda Dagsuyu, Paige Kies, Melissa J. Karau, Robin Patel, Haluk Beyenal

**Affiliations:** 1 School of Chemical Engineering and Bioengineering, Voiland College of Engineering and Architecture, Washington State University 6760, Pullman, Washington, USA; 2 Department of Chemistry, Faculty of Engineering, Istanbul University-Cerrahpaşa 206543, Istanbul, Türkiye; 3 Division of Clinical Microbiology, Mayo Clinic 6915, Rochester, Minnesota, USA; 4 Division of Public Health, Infectious Diseases, and Occupational Medicine, Department of Medicine, Mayo Clinic 6915, Rochester, Minnesota, USA; Icahn School of Medicine at Mount Sinai, New York, New York, USA

**Keywords:** biocide, biofilms, e-bandage, hydrogel, hypochlorous acid, wound healing, methicillin-resistant *Staphylococcus aureus*, *Acinetobacter baumannii*

## Abstract

Biofilms hinder wound infection healing, making wound infections a healthcare challenge for which innovative treatments are needed. A 1.77 cm^2^ electrochemical bandage (e-bandage), which generates the biocide hypochlorous acid (HOCl), has shown promising anti-biofilm activity *in vitro*, *ex vivo*, and *in vivo*. The e-bandage is a three-electrode system operationalized by a hydrogel. In addition to providing a conductive electrolyte for the electrodes, the hydrogel provides a protective barrier for the wound. How different hydrogels impact e-bandage functionality is not completely defined. Here, the *in vitro* activity of seven clinically available hydrogels (xanthan gum, 3M, Duoderm, Prontosan, Purilon, Skintegrity, and Solosite) was evaluated with 1.77 cm^2^ HOCl-generating e-bandages using a potentiostat with a multiplexer in a membrane biofilm model against methicillin-resistant *Staphylococcus aureus* IDRL-6169 and *Acinetobacter baumannii* ATCC-17978. When applied with polarized HOCl-producing e-bandages, all evaluated hydrogels except Solosite yielded ≥5.5 log_10_CFU/cm^2^ reductions in bacterial quantities after 6 h of treatment, with most being below the detection limit at that time point. Prontosan exhibited antibacterial activity independent of e-bandage. Results of this study inform the selection of clinically available hydrogels that may be suitable for use with a 1.77 cm^2^ HOCl-producing e-bandage.

**IMPORTANCE:**

In this study, it was shown that some clinically available hydrogels, combined with a 1.77 cm^2^ HOCl-generating electrochemical bandage, deliver time-dependent antimicrobial activity against methicillin-resistant *Staphylococcus aureus* and *Acinetobacter baumannii* biofilms, offering a potential approach to treat wound infections, including those caused by antimicrobial-resistant bacteria.

## INTRODUCTION

Skin, the body’s largest organ, accounts for ~16% of the body’s total weight (1) and serves as the first line of defense, shielding the body from toxins, microorganisms, and environmental chemicals. Wounds arise when this protective barrier is compromised; chronic wounds form when there is a delay in healing of damaged skin—in this suspended healing state, a chronic wound becomes vulnerable to infection, which further impedes skin repair (2). Beyond infection, chronic wounds heal slowly due to reduced blood flow, persistent inflammation, and impaired re-epithelialization (3). A large portion of chronic wounds harbor biofilms, a barrier to successful wound healing (4). Chronic wound microbiota consists of diverse microorganisms, including aerobic and anaerobic gram-positive and -negative bacteria, as well as fungi. Common pathogens responsible for wound infections include *Enterococcus faecium*, *Staphylococcus aureus*, *Klebsiella pneumoniae*, *Acinetobacter baumannii*, *Pseudomonas aeruginosa*, and *Enterobacter* species (5).

Biofilm-associated wound infections can be tenacious and hard to treat, posing a financial burden on healthcare systems (6). These infections are challenging to treat because the biofilm structure and environment enable pathogens to survive. Conventional treatment often involves antibiotics, increasing the chances of selecting antibiotic-resistant bacteria (7–9). Non-antibiotic approaches using agents such as antimicrobial peptides, surfactants, fatty acids, and metal chelators have been developed to prevent and treat biofilms (10–14). However, the translation of these technologies to the clinic is sometimes difficult (15). New and translatable antimicrobial wound infection prevention, treatment, and healing technologies are needed.

Hypochlorous acid (HOCl) is a reactive oxygen species with potent antimicrobial activity. It is used in clinical medicine for infection control and wound healing (16). It is naturally synthesized by neutrophils as part of the body’s defense against pathogens (17). HOCl kills bacteria by damaging cellular components; HOCl penetrates the cell wall and inhibits DNA and protein synthesis and bacterial growth (18). HOCl also affects bacterial metabolism by decreasing ATP production. This suggests that it would be difficult for bacteria to develop HOCl resistance through exposure to HOCl. Despite its therapeutic potential, a limitation of using HOCl for therapeutic purposes lies in its application. HOCl in liquid form is challenging to apply clinically as it is unstable with decreasing efficacy upon degradation, limiting its use in clinical settings (16). Vashe Wound Cleanser Solution OTC, which contains HOCl, is one approach to address this limitation. Another approach is to use an electrochemical bandage (e-bandage) that provides a continuous supply of freshly generated HOCl at low concentrations.

In previous work, a 1.77 cm^2^ e-bandage, which continuously generates low concentrations of HOCl, was developed and its efficacy shown against mono- and dual-species biofilms, including against *Acinetobacter baumannii*, *Pseudomonas aeruginosa*, and methicillin-resistant *Staphylococcus aureus* (MRSA), *in vitro*, *ex vivo,* and/or *in vivo* (19–25). Lack of selection of resistance after exposure of MRSA and *P. aeruginosa* biofilms to 10 iterations of electrochemically generated HOCl was demonstrated (26), highlighting a minimal risk for selection of HOCl resistance, consistent with its mechanisms of action. The e-bandage is made of carbon fabric working and counter electrodes, as well as a silver-silver chloride (Ag/AgCl) pseudo reference electrode, separated by cotton fabric layers. Electrodes are surrounded by sodium chloride (NaCl) loaded hydrogel, which serves as an electrolyte. The e-bandage working electrode (WE) generates HOCl at a polarization of 1.5 V_Ag/AgCl_ through oxidation of chloride ions (equations 1 and 2) (27).


(1) $$2{Cl}^{-}\Leftrightarrow {Cl}_{2}+2e^{-}\;E^{0}=-1.119V_{Ag/AgCl}$$

(2) $${Cl}_{2}+H_{2}O\Leftrightarrow {Cl}^{-}+HOCl+H^{+}$$

The hydrogel that covers the e-bandage provides electrolytes, enabling ionic conductivity while also maintaining a moist environment (24, 28–30).

To date, the efficacy of the HOCl-generating 1.77 cm^2^ e-bandage has been demonstrated using xanthan gum and, in a limited way, 3M hydrogel (21, 23, 24, 31, 32). For clinical applications, use of clinically available hydrogels beyond the latter may be desired. The goal of this study was to identify hydrogels compatible with the 1.77 cm^2^ HOCl-generating e-bandage in terms of *in vitro* activity against biofilms formed by clinically relevant antimicrobial-resistant bacteria. This study evaluated seven clinically available hydrogels (xanthan gum, 3M, Duoderm, Prontosan, Purilon, Skintegrity, and Solosite), with the addition of 0.9% NaCl for increased ionic conductivity. Activity of 1.77 cm^2^ HOCl-generating e-bandages against MRSA IDRL-6169 and *A. baumannii* ATCC-17978 biofilms was tested with the seven hydrogels.

## MATERIALS AND METHODS

### Chemicals and hydrogels

Analytical-grade compounds from J. T. Baker, Sigma-Aldrich, and Fisher Scientific were utilized. 3M (91111, 3M, USA), Duoderm (187987, Convatec, UK), Prontosan (400517, Bbraun Medical Inc., Germany), Purilon (3903, Coloplast, Denmark), Skintegrity (MSC6201, Medline Industries, USA), and Solosite (449600, Smith & Nephew Medical Limited, USA) hydrogels were studied. Xanthan gum (Namaste Foods, Amazon.com, UPC: 301155217160), used in prior studies, was included as a control hydrogel (21, 23, 24). In addition, prior results with 3M hydrogel were compared to those generated here (32).

### e-bandage construction

**Fig 1:**
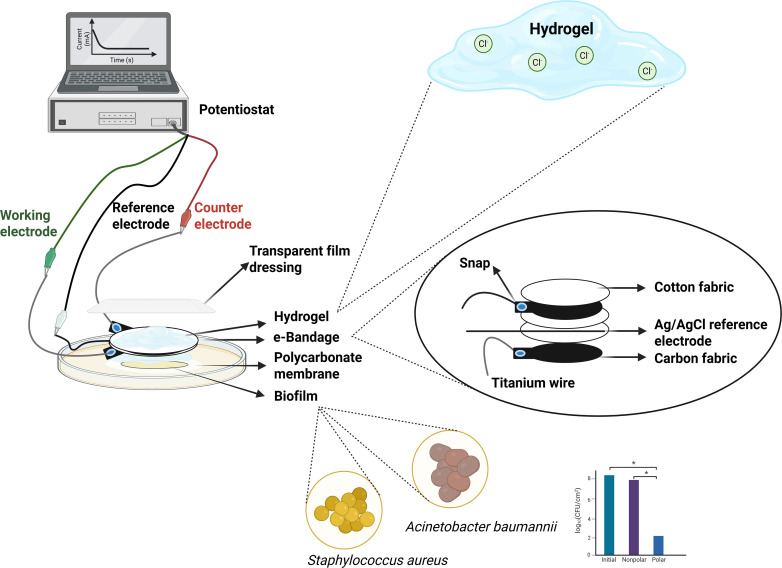
Schematic representation of the 1.77 cm² e-bandage and efficacy experiments. The *in vitro* experimental setup comprised e-bandages interfaced with a potentiostat, applied to laboratory-grown biofilms on tryptic soy agar plates. “Hydrogel” refers to 3M, Duoderm, Prontosan, Purilon, Skintegrity, and Solosite or xanthan gum hydrogels. (Created with BioRender.com.)

The experimental setup is illustrated in Fig. 1. Construction, application, and details of 1.77 cm^2^ e-bandages have been described (25, 29, 33, 34 ). Briefly, the e-bandage is an electrochemical system comprised of three electrodes: two carbon fabric layers serve as the WE and counter electrode (CE) (1.77 cm², Panex 30 PW-06, Zoltek Companies, Inc.), whereas a Ag/AgCl wire functions as a quasi-reference electrode. The electrodes are separated by three layers of cotton fabric (2.25 cm² each) and secured using silicone adhesive (GE Silicone, 30241016, Henkel, USA). Electrical connections are established with 30 AWG titanium wires (TEMco, Amazon.com, catalog no. RW0517), pressed using nylon sew-on snaps (Dritz, Spartanburg, SC, item no. 85), and linked to a potentiostat (Interface 1000E, Gamry) through a multiplexer (ECM8-11012, Gamry). To ensure electrochemical connectivity, the WE and CE are embedded with a hydrogel containing 0.9% NaCl. Prior to use, e-bandages are soaked in phosphate-buffered saline (PBS) for ~15 minutes, followed by the addition of 100 µL of hydrogel containing 0.9% NaCl between the electrode layers to ensure hydration and contact of all components.

### Integration of hydrogels into e-bandages

NaCl (0.76 g) was added per 100 g of each hydrogel; 1× PBS solution was prepared by dissolving Na_2_HPO_4_ (0.01 M), KH_2_PO_4_ (0.0018 M), NaCl (0.137 M), and KCl (0.0027 M) into 18 Ω.m DI water (1 L). Xanthan gum hydrogel was made from 1× PBS mixed with xanthan gum (1.8% wt/vol). Hydrogels and PBS were autoclaved at 121°C for 15 min under liquid cycle conditions.

### *In vitro* agar membrane biofilm model

An established agar-based membrane biofilm model was used, as described (21, 22, 28). For biofilm cultivation, MRSA IDRL-6169 and *A. baumannii* ATCC 17978 were first streaked for isolation from −80°C stocks and incubated at 37°C for 24 h. Two colonies were used to inoculate 2 mL of tryptic soy broth and incubated at 37°C, with shaking at 150 rpm, until cultures reached a turbidity equivalent to a 0.5 McFarland standard. Subsequently, 2.5 µL of the culture was spotted onto the center of a UV-sterilized 13 mm polycarbonate membrane (Whatman Nuclepore hydrophilic membranes, Cytiva, cat. no. 10417001) placed on the surface of a tryptic soy agar (TSA) plate. Inoculated membranes were incubated at 37°C for 24 h. Following this incubation period, the “initial” bacterial load was quantified and recorded as initial colony-forming units per square centimeter (CFU/cm²). The initial value served as a baseline for comparing the activity of subsequent treatments.

### e-bandage treatment

e-bandage treatment was performed on the established membrane biofilm after being transferred to fresh TSA plates. A 100 µL aliquot of the desired hydrogel was injected between the e-bandage cotton fabric layers at the location of the reference electrode using a needle and a syringe. An additional 100 µL of hydrogel was applied directly onto the biofilm, followed by placement of the e-bandage with the WE side in contact with the biofilm. A final 100 µL layer of hydrogel was added on top of the e-bandage. The assembly was covered with a sterile Tegaderm transparent film dressing (3M, reference no. 1622W), and e-bandage wires secured to the side of the TSA plate. Each e-bandage was connected to a potentiostat (Interface 1000E, Gamry) equipped with the multiplexer (ECM8-11012, Gamry). The WE was polarized at +1.5 V_Ag/AgCl_ to allow the generation of HOCl at the WE surface. A nonpolarized condition (no production of HOCl) was used with the same setup, but without electrode polarization. Treated biofilms (i.e., those with polarized e-bandages) were exposed to electrochemically generated HOCl for 1.5, 3, or 6 h.

### Biofilm quantification

Following treatment, e-bandages were placed in sterile petri dishes, and biofilms were removed from the WE surface by flushing the WE surface with 5 mL of PBS. The PBS and membranes were transferred to 15 mL centrifuge tubes, vortexed for 2 min, and sonicated for 5 min at 50 Hz to dislodge and disaggregate biofilm-associated cells. The resulting sonicate fluids were centrifuged at 2,910 × *g* for 10 min, and the pellets were resuspended in 1 mL of PBS. Serial 10-fold dilutions of the resuspension were prepared, and 10 µL of each was spot-plated onto TSA plates. The remaining sonicate fluid (900 µL) was plated onto a single TSA plate. Plates were incubated at 37°C for 24 h, after which the CFUs were counted. Results were expressed as log_10_ CFU/cm² (35). The limit of detection was 0.22 log_10_ CFU/cm² and defined as the quantity resulting in two colonies on the plate inoculated with 900 µL of the undiluted sample; if fewer than two colonies were observed, the results were reported at 0.1 log_10_ CFU/cm².

### Electrochemical activity measurements

Chronoamperometric data were collected from 1.77 cm² e-bandages integrated with the hydrogels while they were polarized using a potentiostat (Interface 1000E, Gamry) equipped with a multiplexer (ECM8-11012, Gamry) at a constant potential of +1.5 V_Ag/AgCl_. After the e-bandage treatment setup was in place, the measurements were conducted using the same e-bandages applied to the biofilms. Data were recorded while the e-bandages remained in contact with biofilms during polarization to assess electrochemical activity under treatment conditions. This approach was chosen because the presence of a biofilm beneath an e-bandage might influence measured current response; therefore, this approach provides more relevant information for assessing the electrochemical behavior of e-bandages during biofilm treatment.

### Statistical analysis

Data were presented as individual data points representing at least four biological replicates (i.e., results of experiments performed on different days), with error bars indicating standard deviations. Group comparisons were conducted using the Wilcoxon rank sum test, and non-parametric tests were selected. All tests were two-tailed; statistical significance was defined as *P* < 0.05. Statistical analyses and figure generation of the data were performed using GraphPad Prism software (version 10.4.1, GraphPad Software).

## RESULTS

*In vitro* activity of the hydrogels paired with HOCl-generating 1.77 cm² e-bandages was assessed against MRSA IDRL-6169 and *A. baumannii* ATCC-17978.

**Fig 2:**
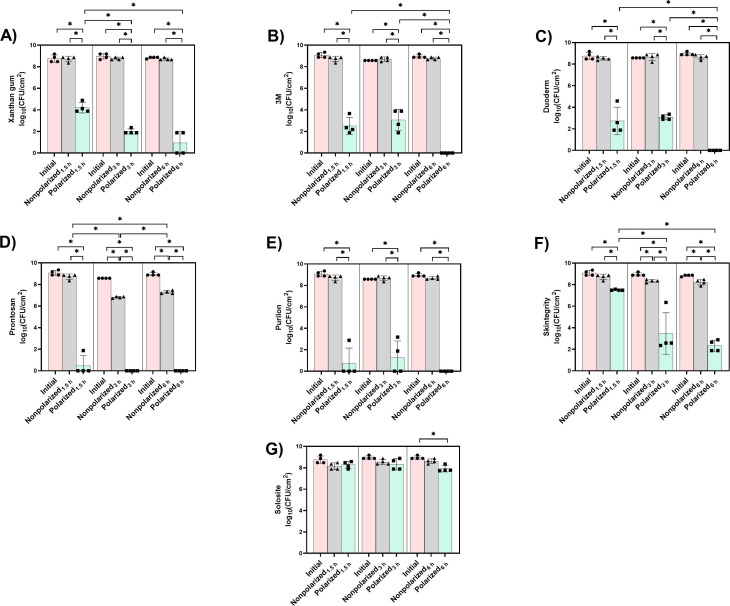
Hydrogels (**A**) xanthan gum, (**B**) 3M, (**C**) Duoderm, (**D**) Prontosan, (**E**) Purilon, (**F**) Skintegrity, and (**G**) Solosite hydrogels tested with 1.77 cm² HOCl-generating e-bandages against MRSA IDRL-6169. Polarized groups (green bars and square symbols) had e-bandages polarized for 1.5, 3, and 6 h. Nonpolarized groups (gray bars and triangle symbols) had e-bandages applied but not polarized for 1.5, 3, and 6 h. Initial groups (pink bars and circle symbols) are initial bacterial quantities (i.e., time zero). Data points represent individual biological replicates (circles, triangles, and squares) and means (horizontal lines) with standard deviations of four independent biological replicates, with statistically significant differences shown as *P < 0.05 (two-sided Wilcoxon rank-sum test).

Figure 2 shows the activities of 1.77 cm² HOCl-generating e-bandages with the hydrogels and a control, xanthan gum hydrogel, against MRSA IDRL-6169 biofilms over 1.5, 3, and 6 h. There was no significant reduction in bacterial load in the nonpolarized e-bandage groups compared with the initial groups, using xanthan gum hydrogel alone, showing that xanthan gum hydrogel lacks inherent antimicrobial activity against MRSA (Fig. 2A). Polarized HOCl-generating e-bandages loaded with xanthan gum hydrogel exhibited a time-dependent decrease in bacteria. After 1.5 h, a 4.5 log_10_ CFU/cm² reduction was observed (*P* < 0.05 vs. initial and nonpolarized groups), progressing to a 6.8 log_10_ CFU/cm² reduction at 3 h (*P* < 0.05 vs. initial and nonpolarized groups) and a 7.8 log_10_ CFU/cm² reduction at 6 h (*P* < 0.05 vs. initial and nonpolarized groups); 3 and 6 h treatments showed significant decreases compared with the 1.5 h treatment (*P* < 0.05). Overall, there was antimicrobial activity of the polarized e-bandage loaded with xanthan gum hydrogel against MRSA biofilms at all time points, with 6 h of treatment approaching the limit of detection.

3M hydrogel is an amorphous hydrogel containing propylene glycol, guar gum, and sodium tetraborate designed to fill wound cavities and provide a moist healing environment (36). Propylene glycol, a humectant, can moisturize wound beds (37). When 3M hydrogel was used with the nonpolarized e-bandages, it showed no significant reduction in bacterial load compared with the initial groups (Fig. 2B). However, polarized HOCl-generating e-bandages showed decreases in bacteria; at 1.5 and 3 h, treated biofilms experienced similar, significant decreases in bacterial load, with 6.2 and 5.6 log_10_ CFU/cm² reductions observed compared with nonpolarized, respectively (*P* < 0.05 vs. initial and nonpolarized groups). This progressed to a reduction of bacteria by 6 h, with bacteria below the limit of detection (*P* < 0.05 vs. initial and nonpolarized groups), with the 6 h treatment showing significant decreases in bacterial load compared with 1.5 and 3 h treatment conditions (*P* < 0.05). This suggests that 3M hydrogel, when combined with polarized HOCl-generating e-bandages, provides effective biocidal activity and is suitable for use with the e-bandage.

Duoderm hydrogel contains a carboxymethylcellulose base and pectin and is designed to provide a moist wound environment (38). The nonpolarized e-bandage groups employing Duoderm hydrogel exhibited no significant reduction in bacterial load compared with the initial groups, indicating that Duoderm hydrogel alone does not possess antimicrobial activity against MRSA DIRL-6169 biofilms (Fig. 2C). When the polarized HOCl-generating e-bandage incorporated Duoderm hydrogel, there was a significant reduction in bacterial burden. At 1.5 and 3 h, 5.8 and 5.6 log_10_ CFU/cm² reductions in bacterial load were observed, respectively (*P* < 0.05 vs. initial and nonpolarized groups); bacteria were reduced to below the limit of detection after 6 h of treatment (*P* < 0.05 vs. initial and nonpolarized groups). These findings show activity of the polarized HOCl-generating e-bandage when combined with Duoderm hydrogel, resulting in rapid and significant reduction of MRSA biofilms over 6 h.

Prontosan hydrogel consists of glycerol, hydroxyethylcellulose, betaine, and polyaminopropyl biguanide (39). Betaine is a surfactant that can modify bacterial surfaces, thereby potentially facilitating the removal of biofilms and wound debris (40). Polyaminopropyl biguanide is an antimicrobial agent with activity against both gram-positive and -negative bacteria, as well as some fungal species (41). Prontosan hydrogel is proposed to provide wound bed cleansing, moistening, and decontamination activities (39). Unsurprisingly, Prontosan hydrogel alone exhibited antimicrobial activity against MRSA biofilms (Fig. 2D); specifically, the nonpolarized e-bandage groups showed a modest, time-dependent reduction in bacterial load at 3 and 6 h (*P* < 0.05 vs. initial) compared with 1.5 h (*P* < 0.05). At 3 and 6 h, the nonpolarized groups showed an ~1.8 log_10_ CFU/cm² reduction compared with the initial bacterial load (*P* < 0.05). Prontosan hydrogel-loaded polarized e-bandages showed a substantial reduction of bacterial burden across the 1.5, 3, and 6 h time points, when compared with the nonpolarized groups (*P* < 0.05). These findings indicate that the polarized HOCl-generating e-bandage, when used with Prontosan hydrogel, results in reduction of MRSA biofilms to below the limit of detection by 3 h. Both polarized and nonpolarized groups showed a greater reduction in bacterial load at 3 and 6 h when compared with initial amounts (*P* < 0.05). This rapid and substantial reduction in bacterial load highlights the additive effect of Prontosan hydrogel with the polarized HOCl-generating e-bandage against MRSA biofilms.

Purilon hydrogel contains sodium carboxymethylcellulose and calcium alginate, designed to provide a moist wound healing environment and intended for use on necrotic and sloughy wounds, leg ulcers, pressure injuries, and non-infected diabetic foot ulcers, in addition to other applications (42). The nonpolarized e-bandage using Purilon hydrogel did not exhibit a significant reduction in bacterial load (Fig. 2E). Use of Purilon hydrogel with the polarized HOCl-generating e-bandage resulted in a marked decrease in bacterial burden, with significant reductions observed at 1.5 (8.0 log_10_ CFU/cm²) and 3 h (7.4 log_10_ CFU/cm²), and both reduced below the limit of detection of the assay, with a greater effect than the nonpolarized groups (*P* < 0.05). These findings indicate that a 6 h polarized HOCl-generating e-bandage using Purilon hydrogel has suitable activity against MRSA biofilms.

Skintegrity hydrogel is composed of allantoin, hydroxyethylcellulose, and dextran (43). Allantoin helps maintain a moist wound environment (44). Hydroxyethylcellulose functions as a thickening, stabilizing, and emulsifying agent (45). Dextran enhances moisture retention, supports biocompatibility, and serves as a structural scaffold (46). As illustrated in Fig. 2F, nonpolarized e-bandage groups utilizing Skintegrity hydrogel showed antimicrobial effects against MRSA biofilms, particularly at 3 and 6 h treatment times, when compared with initial bacterial loads (*P* < 0.05). Additionally, the use of Skintegrity hydrogel with a polarized HOCl-generating e-bandage resulted in a substantial, time-dependent decrease of bacterial burden throughout the treatment duration. A 1.2 log_10_ CFU/cm² reduction was found after 1.5 h of treatment, with more significant reductions with 3 h (4.9 log_10_ CFU/cm² reduction) and 6 h (5.9 log_10_ CFU/cm² reduction) treatments compared with nonpolarized groups (*P* < 0.05). At the 6 h treatment time, bacteria were still detectable. This suggests that although Skintegrity hydrogel supports the HOCl-generating e-bandage function, it is inferior to the 3M, Duoderm, Prontosan, and Purilon hydrogels at the 6 h treatment time (*P* < 0.05).

Solosite is a hydrogel wound dressing consisting of glycerol, sodium carboxymethylcellulose, allantoin, benzyl alcohol, methylparaben, and propylparaben. It is a viscous gel proposed to provide hydration and facilitate autolytic debridement of wounds, including pressure ulcers, leg ulcers, and minor burns (47). The nonpolarized e-bandage groups used with Solosite hydrogel did not exhibit significant antimicrobial activity at the 1.5, 3, and 6 h treatment periods compared with the initial groups (Fig. 2G). Furthermore, the only time point at which the Solosite hydrogel-loaded polarized HOCl-generating e-bandage groups experienced a significant decrease in microbial load compared with the initial was at the 6 h treatment time point (*P* < 0.05). It was concluded that Solosite hydrogel is not well-suited for use with the tested HOCl-generating e-bandage.

**Fig 3:**
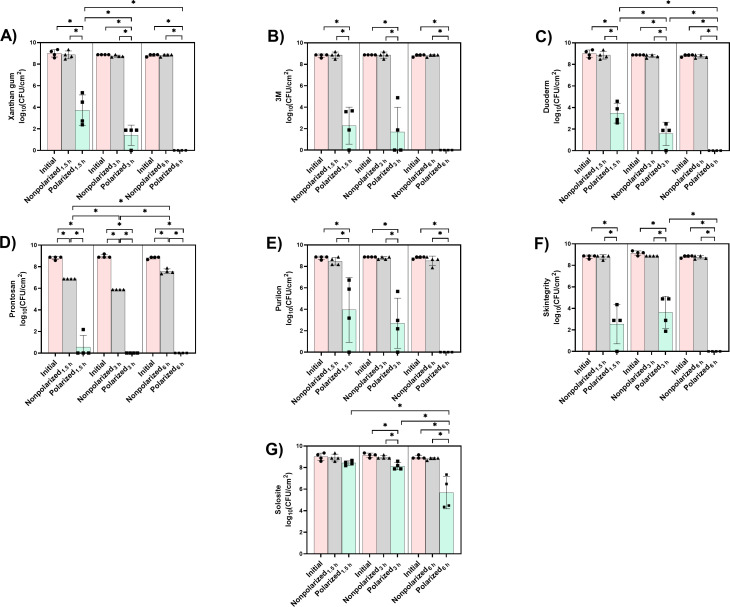
Hydrogels (**A**) xanthan gum, (**B**) 3M, (**C**) Duoderm, (**D**) Prontosan, (**E**) Purilon, (**F**) Skintegrity, and (**G**) Solosite hydrogels) tested with 1.77 cm² HOCl-generating e-bandages against *A. baumannii* ATCC-17978. Polarized groups (green bars and square symbols) had e-bandages polarized for 1.5, 3, and 6 h. Nonpolarized groups (gray bars and triangle symbols) had e-bandages applied but not polarized for 1.5, 3, and 6 h. Initial groups (pink bars and circle symbols) are initial bacterial quantities (i.e., at time zero). Data points represent individual biological replicates (circles, triangles, and squares) and means (horizontal lines) with standard deviations of four independent biological replicates, with statistically significant differences shown as *P < 0.05 (two-sided Wilcoxon rank-sum test).

Figure 3 shows the activity of 1.77 cm² HOCl-generating e-bandages with clinically available hydrogels and xanthan gum hydrogel, against *A. baumannii* ATCC-17978 biofilms over 1.5, 3, and 6 h.

In the xanthan gum hydrogel-loaded nonpolarized e-bandage groups, no significant antimicrobial effect against *A. baumannii* was observed at any of the treatment times (1.5, 3, and 6 h, Fig. 3A). In contrast, xanthan gum-loaded polarized e-bandage groups showed significant, time-dependent reductions in bacterial burden. Bacterial loads were diminished by 5.1 log_10_ CFU/cm^2^ at 1.5 h and 7.3 log_10_ CFU/cm^2^ at 3 h and reduced below the limit of detection at 6 h, when compared with nonpolarized groups (*P* < 0.05 vs. initial and nonpolarized). Furthermore, 3 and 6 h treatments displayed greater reductions in bacterial load compared with 1.5 h treatment (*P* < 0.05). These findings indicate that the polarized HOCl-generating e-bandage used with xanthan gum hydrogel shows substantial and time-dependent reductions in bacterial load.

When e-bandages were paired with 3M hydrogel, the bacterial burden remained consistently high in both the initial and nonpolarized groups across all time points (1.5, 3, and 6 h), indicating that the 3M hydrogel alone does not have antimicrobial effect against *A. baumannii* biofilms (Fig. 3B). In contrast, the polarized HOCl-generating e-bandage showed a significant reduction in bacterial burden. At 1.5 h, the polarized group showed a 6.6 log_10_ CFU/cm^2^ reduction in bacterial load compared with both the initial and nonpolarized groups (*P* < 0.05). This reduction became more pronounced at 3 h (7.2 log_10_ CFU/cm^2^ reduction in bacterial load) compared with initial and nonpolarized groups (*P* < 0.05). After 6 h of polarized treatment, bacterial counts were reduced below the limit of detection (*P* < 0.05 vs. initial and nonpolarized groups). These results show that 3M hydrogel alone does not impact bacterial viability and that its application with the HOCl-generating e-bandage leads to a profound reduction in *A. baumannii* burden over time.

Nonpolarized e-bandages used with Duoderm hydrogel showed no significant reduction in bacterial load against *A. baumannii* biofilms compared with the initial groups (Fig. 3C). This indicates that Duoderm hydrogel alone does not possess inherent antimicrobial activity against *A. baumannii* biofilms under the tested conditions. In contrast, HOCl-generating e-bandages paired with Duoderm hydrogel exhibited a time-dependent decrease in bacterial burden. At 1.5 h, a statistically significant reduction in bacterial load (5.4 log_10_ CFU/cm)^2^ was observed in the polarized group compared with both the initial and nonpolarized groups (*P* < 0.05). A more pronounced decline was found at 3 h, where polarized treatment reduced bacterial load by 7.2 log_10_ CFU/cm^2^ (*P* < 0.05 vs. initial and nonpolarized groups). After 6 h treatment, the bacterial load was reduced to below the limit of detection (*P* < 0.05 vs. initial and nonpolarized groups). The 3 h HOCl-generating e-bandage with Duoderm hydrogel treatment was more active than the 1.5 h treatment (*P* < 0.05), and the 6 h treatment was more active than the 1.5 and 3 h treatments (*P* < 0.05). This shows that Duoderm hydrogel alone does not impact bacterial viability and that its application with the HOCl-generating e-bandage leads to a profound reduction in *A. baumannii* burden over time.

In the presence of Prontosan hydrogel alone, nonpolarized treatment led to a 1.9, 3.1, and 1.2 log_10_ CFU/cm^2^ reductions in *A. baumannii* biofilms compared with the initial inoculum (Fig. 3D) at 1.5, 3, and 6 h treatment times, respectively (*P* < 0.05). This shows antimicrobial activity attributable to Prontosan hydrogel itself against *A. baumannii*, as with MRSA (Fig. 2D). When Prontosan hydrogel was used with HOCl-generating e-bandages, a dramatic and time-dependent decrease in bacterial load was observed. At 1.5 h, a reduction in bacterial load was achieved compared to the nonpolarized group (*P* < 0.05 ns. initial and nonpolarized groups), with three of the four biological replicates being below the limit of detection. A reduction to below the limit of detection was also observed at 3 and 6 h. These results indicate that Prontosan hydrogel has independent antimicrobial activity against *A. baumannii* biofilms, activity which is greater when paired with an HOCl-generating e-bandage treatment.

Figure 3E illustrates 1.5, 3, and 6 h bacterial loads of *A. baumannii* biofilms treated with Purilon hydrogel-loaded HOCl-generating e-bandages. In the nonpolarized e-bandage groups, no significant reduction in bacterial load was observed compared with initial amounts. Conversely, in the polarized e-bandage groups, a significant decrease in bacterial load was observed over time. At 1.5 h, the polarized group showed an ~4.5 log_10_ CFU/cm^2^ reduction (*P* < 0.05 vs. initial and nonpolarized groups). This reduction became more pronounced at 3 and 6 h, with a 6.1 CFU/cm^2^ reduction compared with nonpolarized, respectively (*P* < 0.05 vs. initial and nonpolarized groups). These results suggest that the polarized HOCl-generating e-bandage, when used with Purilon hydrogel, provides effective antimicrobial activity, leading to the complete reduction against *A. baumannii* biofilms within 6 h.

In the Skintegrity hydrogel-loaded nonpolarized e-bandage groups, there was no antimicrobial effect against *A. baumannii* biofilms with 1.5, 3, and 6 h treatments compared with initial bacterial loads (Fig. 3F). However, the polarized e-bandage groups exhibited a significant decrease in bacterial load at all time points tested. At 1.5 and 3 h, the polarized groups showed ~6.3 and 5.3 log_10_ CFU/cm^2^ reductions compared with the nonpolarized groups, respectively (*P* < 0.05 vs. initial and nonpolarized groups). This reduction became more substantial at 6 h, where bacteria were below the limit of detection (*P* < 0.05 vs. initial and nonpolarized groups). These results indicate that the polarized HOCl-generating e-bandage, when used with Skintegrity hydrogel, provides effective antimicrobial activity.

The nonpolarized e-bandage groups paired with Solosite hydrogel had no significant antimicrobial effect against *A. baumannii* at any of the treatment times when compared with initial bacterial counts (Fig. 3G). At 3 and 6 h, the polarized groups showed ~0.8 and 3.2 log_10_ CFU/cm^2^ reductions compared with nonpolarized groups, respectively (*P* < 0.05), with the 6 h treatment being more active than either 1.5 or 3 h treatments (*P* < 0.05). These results suggest that Solosite hydrogel, when combined with the polarized HOCl-generating e-bandage, exhibits a limited antimicrobial effect against *A. baumannii* biofilms and that this effect is only observable at the 3 and 6 h treatments. The observed effects were less pronounced than those of other hydrogels evaluated.

**Fig 4:**
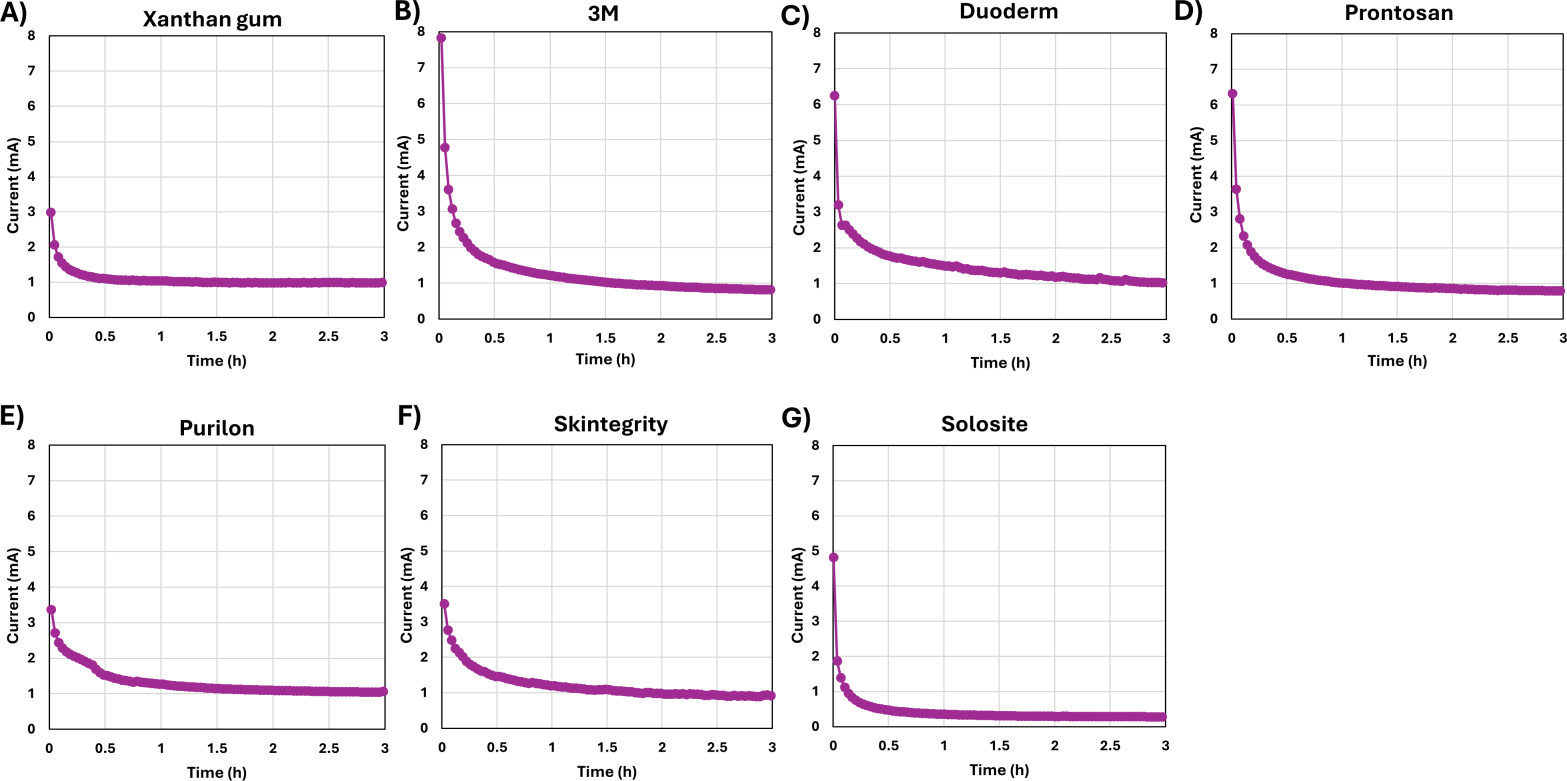
Current-time graphs of (**A**) xanthan gum, (**B**) 3M, (**C**) Duoderm, (**D**) Prontosan, (**E**) Purilon, (**F**) Skintegrity, and (**G**) Solosite hydrogels loaded 1.77 cm^2^ e-bandages polarized at 1.5 V_Ag/AgCl_.

Figure 4 presents chronoamperometric data from e-bandages loaded with xanthan gum (Fig. 4A), 3M (Fig. 4B), Duoderm (Fig. 4C), Prontosan (Fig. 4D), Purilon (Fig. 4E), Skintegrity (Fig. 4F), and Solosite (Fig. 4G) hydrogels polarized at a constant potential of 1.5 V_Ag/AgCl_ over a 3 h duration. e-bandages with most study hydrogels generally retained a stable current response at nearly 1 mA; however, the Solosite hydrogel exhibited a current value under 0.4 mA. Since the only variable among the tested hydrogels was chemical composition, the 60% reduction in anodic current observed with the Solosite hydrogel is likely attributable to additional resistance to electrochemical reactions or ion transfer introduced by the hydrogel. This may account for the reduced antimicrobial activity of Solosite hydrogel when paired with the HOCl-producing e-bandage.

## DISCUSSION

In total, 1.77 cm^2^ HOCl-generating e-bandages showed activity against MRSA and *A. baumannii* biofilms when used with most of the tested clinically available hydrogels, with Solosite hydrogel having the least activity.

Six-hour treatment with HOCl-generating e-bandages with 3M, Duoderm, Prontosan, or Purilon hydrogels showed reductions of MRSA biofilm below the limit of detection. Skintegrity and xanthan gum hydrogel-loaded HOCl-generating e-bandages showed better bacterial reduction against MRSA biofilms than did Solosite-loaded HOCl-generating e-bandages (*P* < 0.05), but skintegrity hydrogel reduced activity compared to 3M, Duoderm, Prontosan, or Purilon hydrogel-loaded HOCl-generating e-bandages (*P* < 0.05). When the activity against MRSA biofilms was evaluated over time, polarized e-bandages loaded with 3M, Duoderm, and Prontosan hydrogels presented time-dependent antimicrobial effects, where 6 h treatment was more active than 1.5 or 3 h treatments. For skin integrity and xanthan gum hydrogels, both 3 and 6 h treatments showed improved activity of the e-bandage compared with the 1.5 h treatment.

Hydrogel-loaded HOCl-generating e-bandages paired with 3M, Duoderm, Prontosan, Purilon, Skintegrity, or xanthan gum for 6 h of treatment showed reduction in *A. baumannii* biofilms to below the limit of detection compared with nonpolarized groups. There was a small, yet significant, decrease in bacterial load after 6 h of treatment with Solosite hydrogel-loaded HOCl-generating e-bandages compared with the nonpolarized group. For *A. baumannii* biofilms, Duoderm paired polarized e-bandages presented time-dependent bacterial load reductions, with 3 h treatments being more active than 1.5 h treatments, and 6 h treatments outperforming 1.5 and 3 h treatments. For Skintegrity and Solosite, 6 h treatments were more active than 3 h treatments, with Solosite presenting a statistically significant improvement at 6 h compared with 1.5 h. For the xanthan gum hydrogel, both 3 and 6 h treatments were more active than the 1.5 h treatment.

All evaluated hydrogels, except Solosite, were found to be potentially suitable for use with HOCl-generating e-bandages. However, variations in e-bandage activity were observed, likely due to differences in the physicochemical properties of each hydrogel. For example, based on the results, it was estimated that more than 6 h of treatment was needed to reduce the entire bacterial load when Solosite hydrogel was used with a polarized e-bandage. It was also noted that Prontosan, but not the other hydrogels (with the exception of a small but statistically significant difference for Skintegrity hydrogel with MRSA biofilms at 3 h), had intrinsic antimicrobial activity; despite this, the activity of Prontosan hydrogel was improved with the HOCl-generating e-bandage.

The efficacy of the clinically available hydrogel-loaded, 1.77 cm^2^ HOCl-generating e-bandages against MRSA and *A. baumannii* is generally consistent with previous studies using a control xanthan gum hydrogel, with exceptions as noted. Further testing is needed to confirm findings with other bacterial and yeast species.

## Data Availability

The data that support the ﬁndings of this study are available from the corresponding author upon request.
